# An unusual cause of shoulder pain: osteochondroma of ventral scapula (a case report)

**DOI:** 10.11604/pamj.2021.39.88.29345

**Published:** 2021-05-28

**Authors:** Ameni Ammar, Oussama Abcha, Akram Zaier, Leila Bouhajja, Faten Farah, Mahmoud Smida, Mohamed Samir Daghfous

**Affiliations:** 1 Traumatology Department, Kassab Institute, Manouba, Tunisia

**Keywords:** Osteochondroma, snapping scapula, winging scapula, case report

## Abstract

Osteochondromas mainly affect the metaphysis of long bones such as femur, humerus, and tibia. It is unusual in flat bones such as scapula. Osteochondroma of ventral surface of scapula is one of the rare cause of shoulder pain and difficult to diagnose in first place. We report the case of an 18-year old girl, presenting progressive right shoulder pain for two years. Physical examination showed an imbalance of the shoulders, a winging of the right scapula, and a snapping of the shoulder on mobilization. Radiographic evaluation showed a pedunculated bony structure extruding from the scapula. Computed tomography (CT) scanner and magnetic resonance imaging (MRI) revealed a bony exostosis along the medial border on the ventral surface of the right scapula. The patient had an excision of the exostosis. Histologic examination confirmed that the specimen was an osteochondroma with no signs of malignant transformation. The shoulder was immobilized for two weeks. The patient has regained full function of her shoulder, six weeks postoperatively.

## Introduction

Osteochondromas constitute 10-15% of all bone tumours and 30-50% of benign bone tumours, representing the most common benign bone tumours [1]. It usually occurs in the metaphysis of long bones such as femur, humerus, and tibia. It is unusual in flat bones such as scapula, seen only in 4% of cases [2]. A scapular osteochondroma may be symptomatic mainly due to its mass effect. Diagnosis may be difficult when it arises from the ventral surface of the scapula and it may be missed on plain radiographs. Due to its location on ventral scapula and atypical presentation of pseudo-winging, diagnosis of osteochondroma of scapula is often delayed. The following case describes a patient with ventral osteochondroma causing shoulder pain, pseudo-winging and snapping scapula syndrome.

## Patient and observation

An 18-year-old right-handed girl, presented to our hospital, complaining pain of right shoulder, for two years, which had been gradual in onset. The pain was radiating to the upper back. The patient was healthy without fever or traumatic injury. There was an associated swelling over the right scapular region. It has gradually increased in size over the past year and has caused a pseudo-wing of the right scapula. She had several consultations in many orthopaedic departments. Because of the shoulders imbalance, scoliosis has been suspected, and the patient has had several spinal imaging exams (spinal X-rays and Medullary MRI), which were normal. Physical examination showed an imbalance of the shoulders with a winging of the right scapula, which was difficult to identify because the patient was obese. Physical examination showed also, a crepitus of the shoulder on mobilization. A full range of motion was found in both shoulders.

**Figure 1:**
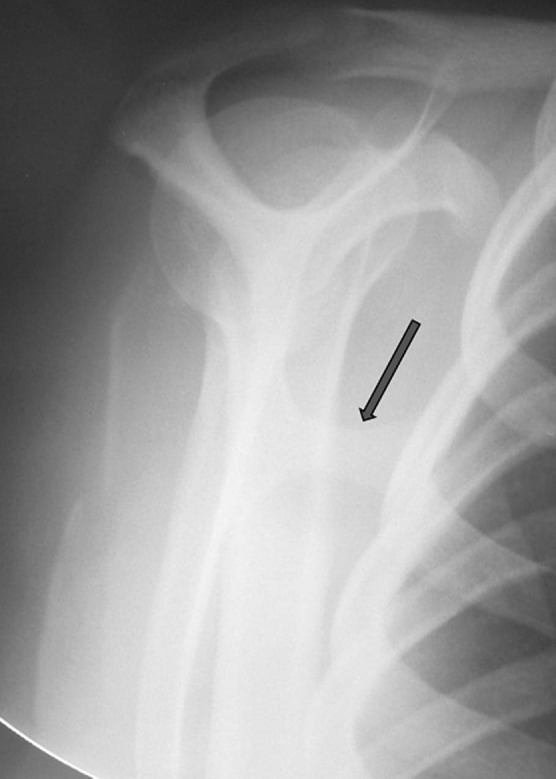
standard radiography showing a pedunculated bony structure extruding from the right scapula

**Figure 2:**
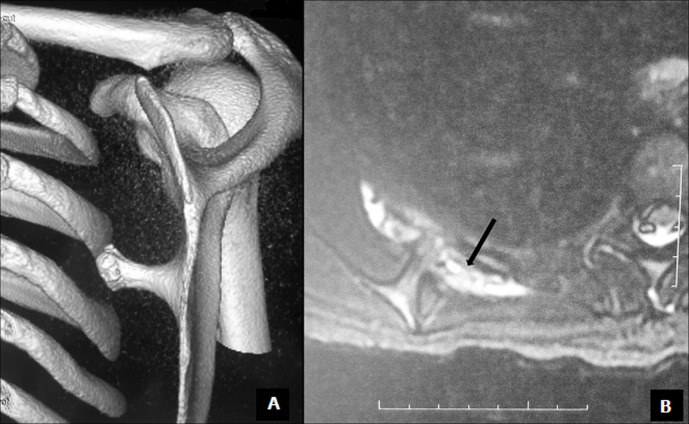
computerized tomography (CT) scanner showing a bony exostosis along the medial border on the ventral surface of the right scapula (A); MRI showing an interscapulo-thoracic bursitis next to the exostosis (B)

**Figure 3:**
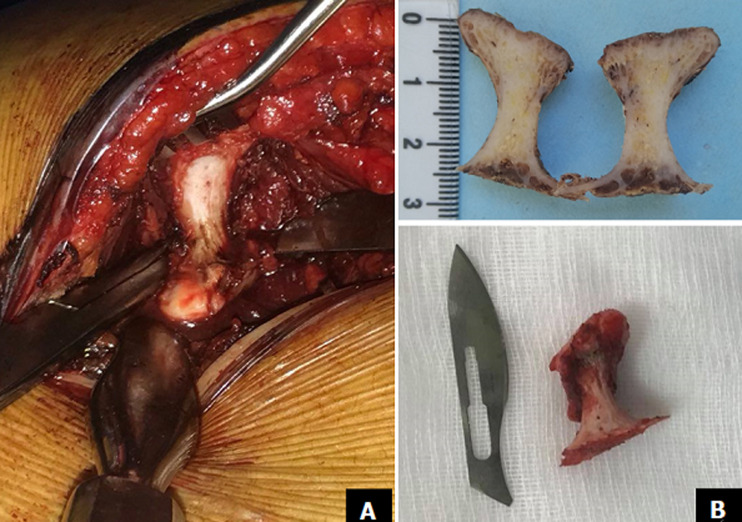
intraoperative photo showing the osteochondroma with the bursitis (A); the osteochondroma was excised at its base (B)

**Figure 4:**
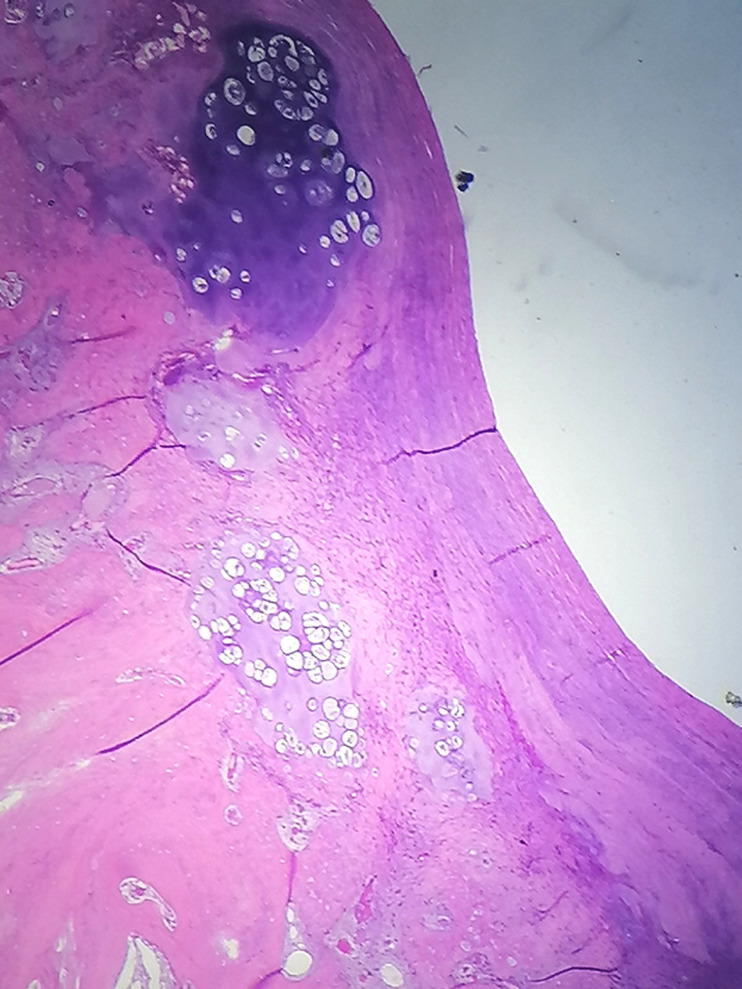
the osteochondroma had no signs of malignant transformation, and was topped by a thin layer of regular cartilage, which was topped by a fibrous layer

Radiographic evaluation showed a pedunculated bony structure extruding from the scapula (Figure 1). CT scanner revealed a bony exostosis along the medial border on the ventral surface of the right scapula (Figure 2A). The MRI showed an interscapulo-thoracic bursitis next to the exostosis (Figure 2B). There were no signs of malignant transformation. The patient was operated in a prone position, with general anaesthesia. Through a parascapular approach along the medial border of the right scapula, we performed an excision of the exostosis at its base (Figure 3). Histologic examination confirmed that the specimen was an osteochondroma with no signs of malignant transformation. The exostosis was topped by a thin layer of regular cartilage, which was topped by a fibrous layer (Figure 4). The pedicle of the exostosis consisted of bony trabeculae. The shoulder was immobilized for two weeks. The patient had no pain, and full range of right shoulder motion at six weeks follow up.

## Discussion

An osteochondroma is a bone exostosis in which a continuous cortical layer projects from the underlying bone. It is filled with cancellous bone and covered by a cartilaginous tissue of 1 to 3 mm in thickness. Most osteochondromas are single lesions, which can occur in two forms: sessile or pedunculated [3]. The most common site of osteochondroma is the metaphysis of tubular long bones, with the distal femur, proximal tibia, and proximal humerus constituting 90% of the occurrence site. Flat bones such as the pelvis and the scapula are relatively rare sites for osteochondroma, with a 3% to 4.5% involvement of the scapula [4,5]. However, it is the most common benign tumour of the scapula, accounting for approximately 5% of such lesions [3]. Osteochondromas are easily diagnosed in the appendicular skeleton. However, atypical localization and malignancy are sometimes challenging to diagnose on clinical evaluation and plain radiography. Therefore, more refine diagnostic tools may be required.

Scapula osteochondroma is usually asymptomatic. However, it may present with features such as chronic pain, oedema, bursa formation, scapular protuberance, crepitation, and loss of mobility. Other symptoms, may be present depending on the size and location of the tumour such as pseudo-winging and snapping scapula [6]. The pseudo-winging of the scapula is observed when the tumour is located in the medial, inferior, and ventral border of the scapula, where the tumour concavity is dislocated over the convex aspect of the rib cage, leading the scapula to the lateral region of the shoulder girdle [6].

The snapping scapula syndrome is an infrequently described cause of shoulder pain. This syndrome, is always accompanied by audible and/or palpable crepitus of the scapula with scapulothoracic motion. It is a tactile-acoustic phenomenon secondary to an abnormality between the anterior surface of the scapula and the thoracic wall. The causes of the snapping scapula syndrome have been classified as abnormalities of the bone, muscle, or bursa that are involved with scapulothoracic movement [7]. It was reported that exostoses of the ventral scapula, has been complicated by chest wall compression in the article of Chun *et al*. [5], and by a pseudoaneurysm of the subclavian artery in the article of Oljaca *et al*. [8]. So, the exostosis of the ventral scapula must be excised surgically in time, before the occurrence of mechanical complications. There are three main surgical approaches to removal of scapula exostosis: muscles sparing, muscle detaching and endoscopically assisted techniques [9]. In our case, we used a muscle sparing technique.

## Conclusion

The diagnosis of osteochondroma of the ventral scapula should be considered in any patient with pain of shoulder region, scapular winging, and snapping during mobilisation. This is important to avoid unnecessary investigations searching and to avoid missing the diagnosis initially. Surgical resection should be performed on time, before the occurrence of mechanical complications.
